# Humanized Mice Recapitulate Key Features of HIV-1 Infection: A Novel Concept Using Long-Acting Anti-Retroviral Drugs for Treating HIV-1

**DOI:** 10.1371/journal.pone.0038853

**Published:** 2012-06-13

**Authors:** Marc Nischang, Roger Sutmuller, Gustavo Gers-Huber, Annette Audigé, Duo Li, Mary-Aude Rochat, Stefan Baenziger, Ursula Hofer, Erika Schlaepfer, Stephan Regenass, Katie Amssoms, Bart Stoops, Anja Van Cauwenberge, Daniel Boden, Guenter Kraus, Roberto F. Speck

**Affiliations:** 1 Division of Infectious Diseases and Hospital Epidemiology, University Hospital of Zurich, University of Zurich, Zurich, Switzerland; 2 Tibotec BVBA, Beerse, Belgium; 3 Clinical Immunology, University Hospital of Zurich, University of Zurich, Zurich, Switzerland; University of California, San Francisco, United States of America

## Abstract

**Background:**

Humanized mice generate a lymphoid system of human origin subsequent to transplantation of human CD34+ cells and thus are highly susceptible to HIV infection. Here we examined the efficacy of antiretroviral treatment (ART) when added to food pellets, and of long-acting (LA) antiretroviral compounds, either as monotherapy or in combination. These studies shall be inspiring for establishing a gold standard of ART, which is easy to administer and well supported by the mice, and for subsequent studies such as latency. Furthermore, they should disclose whether viral breakthrough and emergence of resistance occurs similar as in HIV-infected patients when ART is insufficient.

**Methods/Principal Findings:**

NOD/shi-scid/γ_c_null (NOG) mice were used in all experimentations. We first performed pharmacokinetic studies of the drugs used, either added to food pellets (AZT, TDF, 3TC, RTV) or in a LA formulation that permitted once weekly subcutaneous administration (TMC278: non-nucleoside reverse transcriptase inhibitor, TMC181: protease inhibitor). A combination of 3TC, TDF and TMC278-LA or 3TC, TDF, TMC278-LA and TMC181-LA suppressed the viral load to undetectable levels in 15/19 (79%) and 14/14 (100%) mice, respectively. In successfully treated mice, subsequent monotherapy with TMC278-LA resulted in viral breakthrough; in contrast, the two LA compounds together prevented viral breakthrough. Resistance mutations matched the mutations most commonly observed in HIV patients failing therapy. Importantly, viral rebound after interruption of ART, presence of HIV DNA in successfully treated mice and in vitro reactivation of early HIV transcripts point to an existing latent HIV reservoir.

**Conclusions/Significance:**

This report is a unique description of multiple aspects of HIV infection in humanized mice that comprised efficacy testing of various treatment regimens, including LA compounds, resistance mutation analysis as well as viral rebound after treatment interruption. Humanized mice will be highly valuable for exploring the antiviral potency of new compounds or compounds targeting the latent HIV reservoir.

## Introduction

The HIV pandemic continues to spread. Even in the United States and Europe with their relative universal access to combined anti-retroviral treatment (ART), the prevalence of HIV-infected people is increasing. The 2nd and 3rd generation antivirals are very efficacious. The life expectancy of treated HIV-infected individuals has significantly improved over the last two decades [1] and, in turn, has contributed to the increasing prevalence. However, ART has significant shortcomings, including adverse events, psychological dependence, life-long adherence and cost. Incomplete adherence results in the emergence of drug-resistant HIV strains. Novel and simpler treatment strategies and, in the best-case scenario, a cure are needed.

The HIV pandemic started with an ancestral SIV from a non-human primate crossing into humans [2], and thus, it is not surprising that HIV replication is limited to human and non-human primate cells. Mouse models of HIV infection have been generated by engrafting human lymphoid tissue into SCID mice [3] and are receptive to HIV [4]. For example, SCID mice transplanted with fetal liver (liv) and thymus (thy) tissue were very valuable for studying various aspects of HIV pathogenesis, including HIV-induced pathology in the thy/liv implant, screening anti-viral compounds and hematopoietic stem cell–based gene therapy [5]. However, these studies are limited to the thy/liv implant.

Development of a human adaptive immune system in cord blood cell–transplanted mice [6] renewed the interest in humanized mice (hu mice): hematopoietic CD34+ cells were preferentially transplanted into the liver of newborn mice or i.v. at older age. The mice develop a lymphoid-like system of human origin with T and B cells, monocytes, plasmacytoid and conventional DCs, thymus and lymph nodes [7]. Their mature T cells have a broad Vβ repertoire, and more than 40% of T cells display a naive phenotype [6]. This breakthrough was only realized through the development of heavily immunodeficient mice by crossing of SCID mice with non-obese diabetic (NOD) mice and mice deficient in the gamma c (γ_c_) chain of the IL-2 receptor or the generation of Rag1 or 2−/− γ_c_−/− knock-out mice [8].

We and others demonstrated that these hu mice are highly permissive to HIV infection when challenged with CCR5- or CXCR4-tropic HIV strains and show viral dissemination and progressive CD4+ T-cell loss [9], [10], [11], [12], [13].

To validate the experimental significance of hu mice for studying HIV pathogenesis and their value for novel interventional approaches, key aspects of HIV infection/pathogenesis must be fulfilled. The model should recapitulate ART of disseminated HIV infection with subsequent recovery of the immune system; interruption of ART should result in a rebound of HIV replication from the latent reservoir. Five studies reported the effects of ART in HIV-infected humanized mice using different drugs and drug combinations [14], [15], [16], [17], [18]. While these reports are very promising that humanized mice may be the long awaited small animal model for preclinical proof-of-concept studies, they lack pharmacokinetic (PK) studies for the medicinal compounds used except for the report by Choudhary et al [15] that would help to compare the data obtained in humanized mice to a clinical context.

A “standardized" ART scheme that completely suppresses HIV RNA replication in hu mice would be highly valuable for pre-clinical proof-of-concept studies for novel anti-retroviral compounds and studies of latency that closely approximate the situation in HIV-infected humans treated with ART.

Thus, we sought to determine if this mouse model is valuable for studying antiretroviral treatment of disseminated HIV infection and if it recapitulates key features, such as viral rebound, break-through replication and viral rebound subsequent to interruption of ART. We made a major effort to define the dose of antiretroviral compounds added to the food pellets to compare data to human studies. We also benefited from access to antiretroviral compounds in special formulation that permit once weekly dosing in mice. Thus, we also were able to study a novel concept of anti-HIV therapy based on LA antiretroviral drugs.

## Results

### Engraftment with human cells at around week 15 when hu mice were infected with HIV

Newborn NOG mice were transplanted with CD34+ haematopoietic progenitor cells isolated from umbilical cord blood. At around 15 weeks of age, the engraftment level was 20.7%±13.2 (avg ± std) before HIV infection (Figure S1). Of all human cells, CD4+ T cells were 23.1%±14.5 (avg ± std), CD8+ T cells 12.9%±9, and CD19+ B cells 50.8%±24.2. These engraftment values and their cell subset distributions are similar as reported previously [11], [19], [20].

### Pharmacokinetics of 3TC, Tenofovir (TDF), TMC278-long-acting (LA) and TMC181-LA

The easiest and most convenient way for long-term ART in hu mice would be to add the ART to the food pellets. Mice have a higher metabolism than humans, and thus, we converted the dose of the distinct compounds used in humans by a formula as described [21]. The dose calculated was 61.7 mg/kg/day for 3TC and for TDF, considering food uptake of 3–4 g/d for a hu mouse with a body weight of 20–30 g. We generated food pellets containing 0.5 mg of 3TC and TDF per g of food. 3TC and TDF belong to the group of nucleoside resp. nucleotide reverse transcriptase inhibitors (NRTIs).

**Figure 1:**
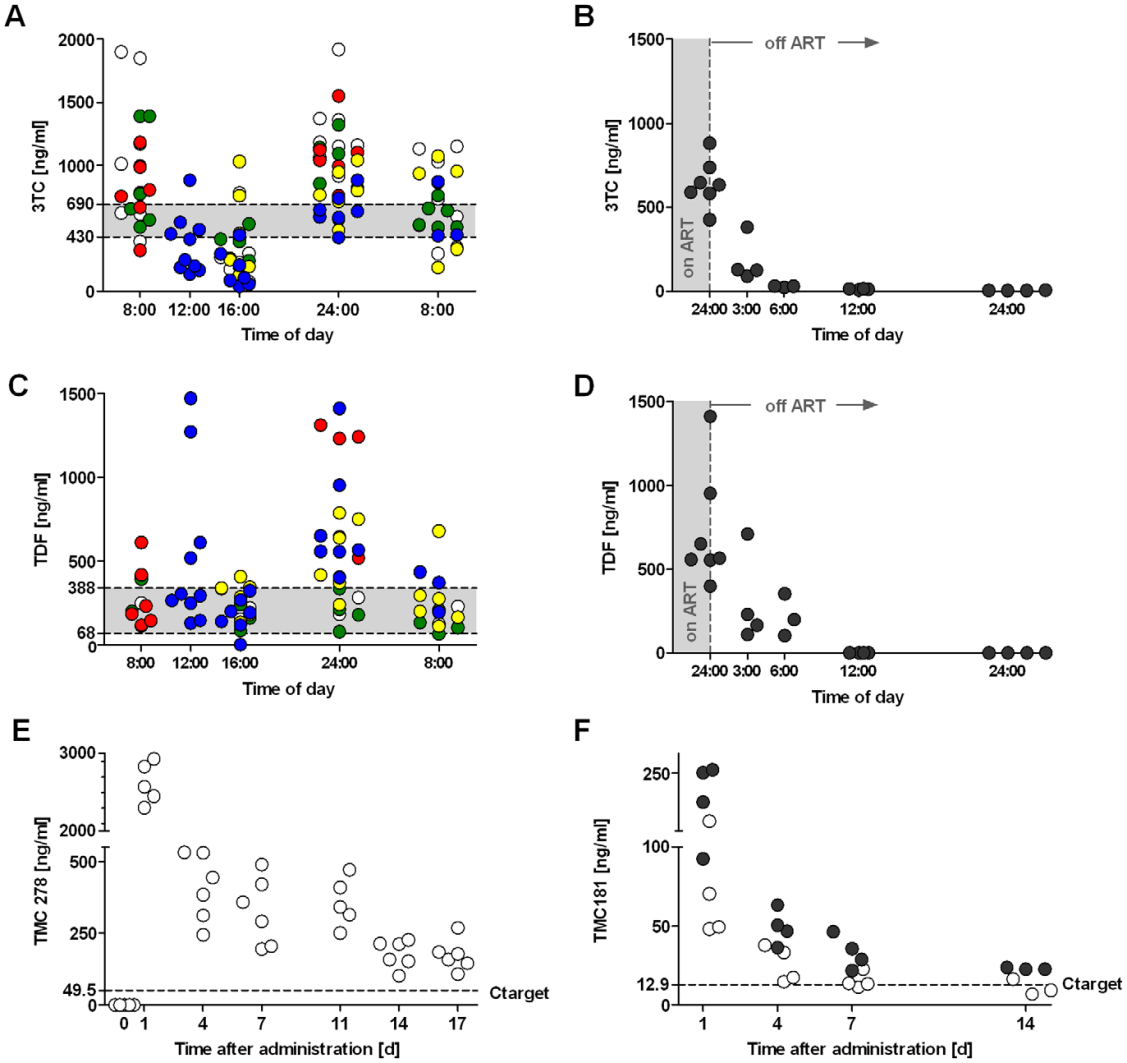
PK data for 3TC, TDF, TMC278-LA and TMC181-LA. (A and C) Plasma levels of 3TC and TDF, respectively, over a day of mice on food pellets containing 0.5 mg/g food of 3TC or TDF for 2 weeks. (B and D) Decay rate of 3TC (t½ = 5.5 h) and TDF (t½ = 3.5 h), respectively, when replacing the food containing 3TC or TDF with standard food. (E and F) Plasma levels after one dose of either TMC278-LA (160 mg/kg) or TMC181-LA (white dots: 200mg/kg; black dots: 400mg/kg) administered s.c. The data were obtained with mice on ART-containing food pellets for at least 2 weeks to permit PK equilibration. The shaded area in (A–D) indicates the therapeutic range as defined in humans [42], [43]. The dashed line in (E and F) indicates the target concentration (C target). Median effective concentration (EC) 50 values of TMC278 and TMC181 are 4.95 ng/ml and 1.29 ng/ml respectively *in vitro* in MT4 cells cultured with 50% human serum. The different colours indicate the experiments done with the same food batch, and whether we used mice transplanted with human CD34+ cells or not (White, red and yellow dots indicate humanized mice, green and blue dots indicate mice without transplantation of human CD34+ cells).

PK data validated this approach showing plasma levels in the therapeutic range over the entire observation period with fluctuations due to the wake-sleep cycle of the mice (Fig. 1A–D). In contrast, azidothymidine (AZT) at 0.5 mg/g of food and ritonavir (RTV) at 1 mg/g of food gave toxic concentrations clearly above the therapeutic range or sub-inhibitory concentrations, respectively (Figure S2). The plasma concentration of TMC278-LA and TMC181-LA (at the higher dose) was still clearly above the target concentration (C_target_) even 14 days after s.c. injection (Fig. 1E–F). TMC278 is the recently approved non-nucleoside reverse transcriptase inhibitor (NNRTI) rilpivirine [22], and TMC181 is a pre-clinical-stage protease inhibitor (PI) belonging to the same chemical class as TMC114 (darunavir), but displaying better potency while preserving similarly high resistance coverage. The PK data of the long-acting drugs permitted a once weekly application in mice.

### HIV RNA plasma level is suppressed by ART and promptly rebounds with treatment interruption

**Figure 2:**
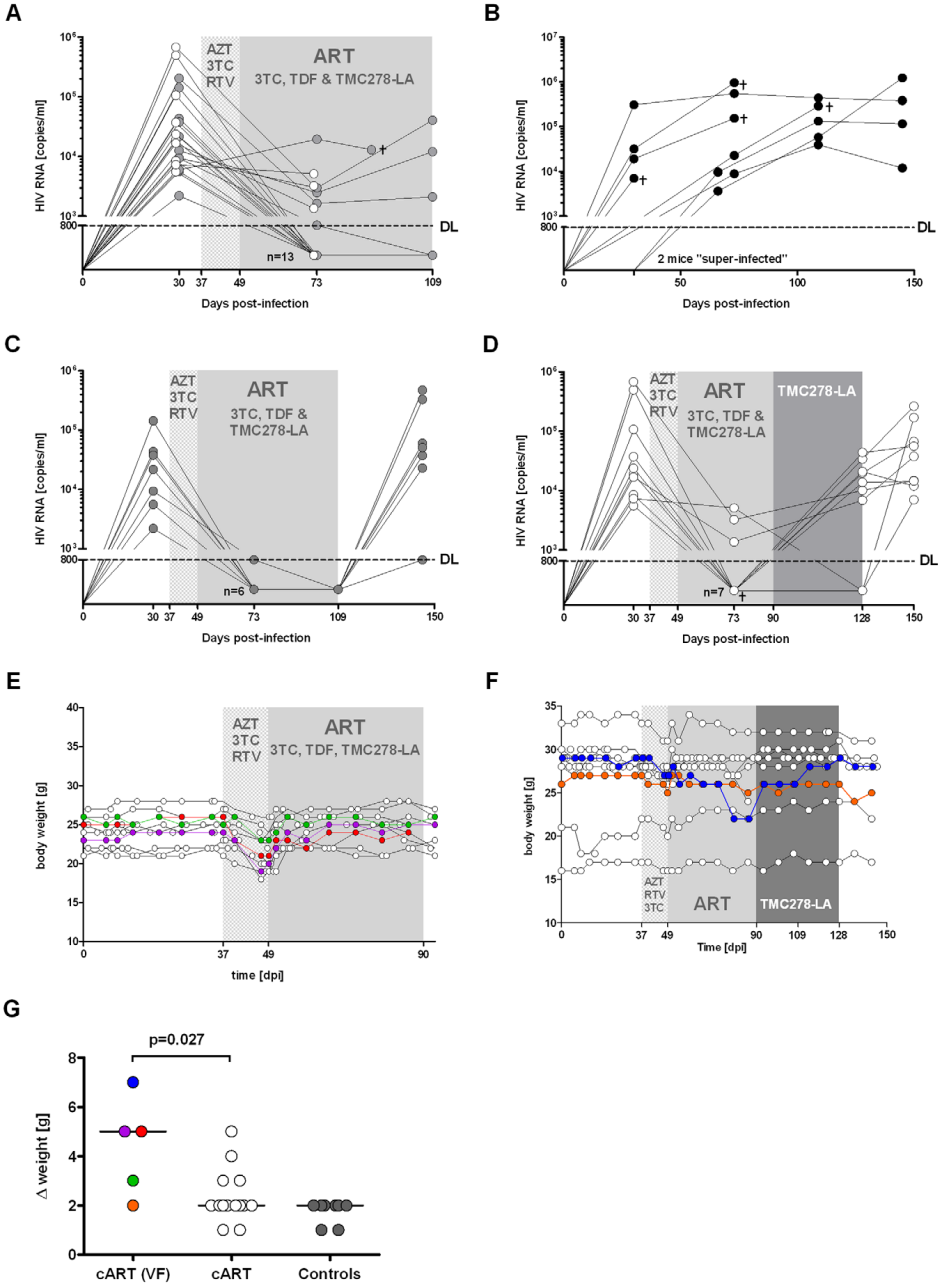
ART is highly efficient in disseminated high-titer HIV infection in hu mice. (A) Response to ART with AZT, 3TC and RTV, followed by 3TC, TDF and TMC278-LA. Note that the initial regimen was poorly tolerated by the hu mice that resulted in reduced food uptake and thus a somewhat lower response rate. (B) Mock-treated HIV-infected mice (n = 8). (C and D) Mice with suppressed HIV RNA were either kept on ART (n = 11) (C) or switched to monotherapy with TMC278-LA alone (n = 10) (D). ART and monotherapy were interrupted to monitor the mice for viral rebound. We included two mice with viral failure into the group treated with TMC-278-LA alone. ┼ indicates that one mouse died within one week of bleeding. (E and F) Weight monitoring of mice either constantly on combined ART (E) or switched subsequently to monotherapy with TMC278-LA (F). Compilation of weight loss over time of all mice, i.e., mice on ART with viral failure (VF), mice on ART with suppressed HIV RNA and untreated HIV-infected mice (controls) (G). Grey-spotted area indicates the time period hu mice were on ART with AZT, 3TC and RTV, grey-plain shaded the time period on ART with 3TC, TDF and TMC278-LA. The coloured circles indicate the mice with viral failure.

In a pilot experiment, uninfected mice appeared to tolerate AZT, 3TC and RTV well. Thus, while awaiting the PK data of these compounds, we started a first experiment to examine ART in HIV-infected mice (Fig. 2). Mice before ART had an HIV RNA baseline of 10^4.9^±10^5.2^ copies/ml (avg ± std). Unexpectedly, the treated mice developed wasting within 2 weeks, which we attributed to the ART and, in particular, AZT. We therefore changed the ART immediately to TDF, 3TC and TMC278-LA, and within 1 week, the mice recovered from the wasting disease. Importantly, within 4–8 weeks, 14/21 (66%) mice showed a decline of HIV RNA levels to under the detection limit of 800 copies/ml (Fig. 2A). One mouse with detectable HIV RNA at that time showed suppressed HIV RNA when we bled it 72 days after start of ART (Fig. 2A). Since we switched the ART in two mice with detectable HIV RNA to monotherapy with TMC278-LA, we are not able to make any statement about their eventual response rate if the ART had been continued. Thus, the overall response rate to the ART (3TC/TDF/TMC278-LA) was 79% (15/19 mice).

In an additional group of mice treated with AZT, 3TC and RTV, we found various laboratory disturbances, most prominent a very significant anemia (Figure S3). In concert with plasma AZT levels clearly above the therapeutic range, we attributed the wasting observed to AZT toxicity.

Nearly all mice receiving the 2 weeks of AZT, 3TC and RTV treatment suffered from weight loss (Fig. 2E and F). Remarkably, the mice with viral failure experienced more significant weight loss than the others (Fig. 2G). Mock-treated HIV-infected mice showed a very stable weight course with fluctuations of less than 2 g over time.

We subsequently divided the mice with suppressed HIV RNA into two groups, seven mice were maintained on ART for another 5 weeks and, thereafter, treatment was interrupted. The other seven mice were treated by TMC278-LA alone. ART interruption resulted in viral rebound in all mice, indicating the existence of a latent reservoir, such as that in HIV-infected humans (Fig. 2C). Monotherapy with TMC278-LA was insufficiently potent to suppress HIV RNA since 6/7 mice showed a breakthrough of viral replication (Fig. 2D).

While there were no obvious symptoms or signs, we observed a higher mortality in mock-treated HIV infected mice than in ART treated mice. We associated this higher mortality with an unknown HIV-associated phenomenon. This mortality was usually less than 20% and thus when working with small numbers of mice, there may be divergence from this estimated mortality rate. In the 2nd set of experiments presented (see below), we had no loss due to “spontaneous" mortality.

### Emergence of drug-resistant HIV strains in hu mice

**Figure 3:**
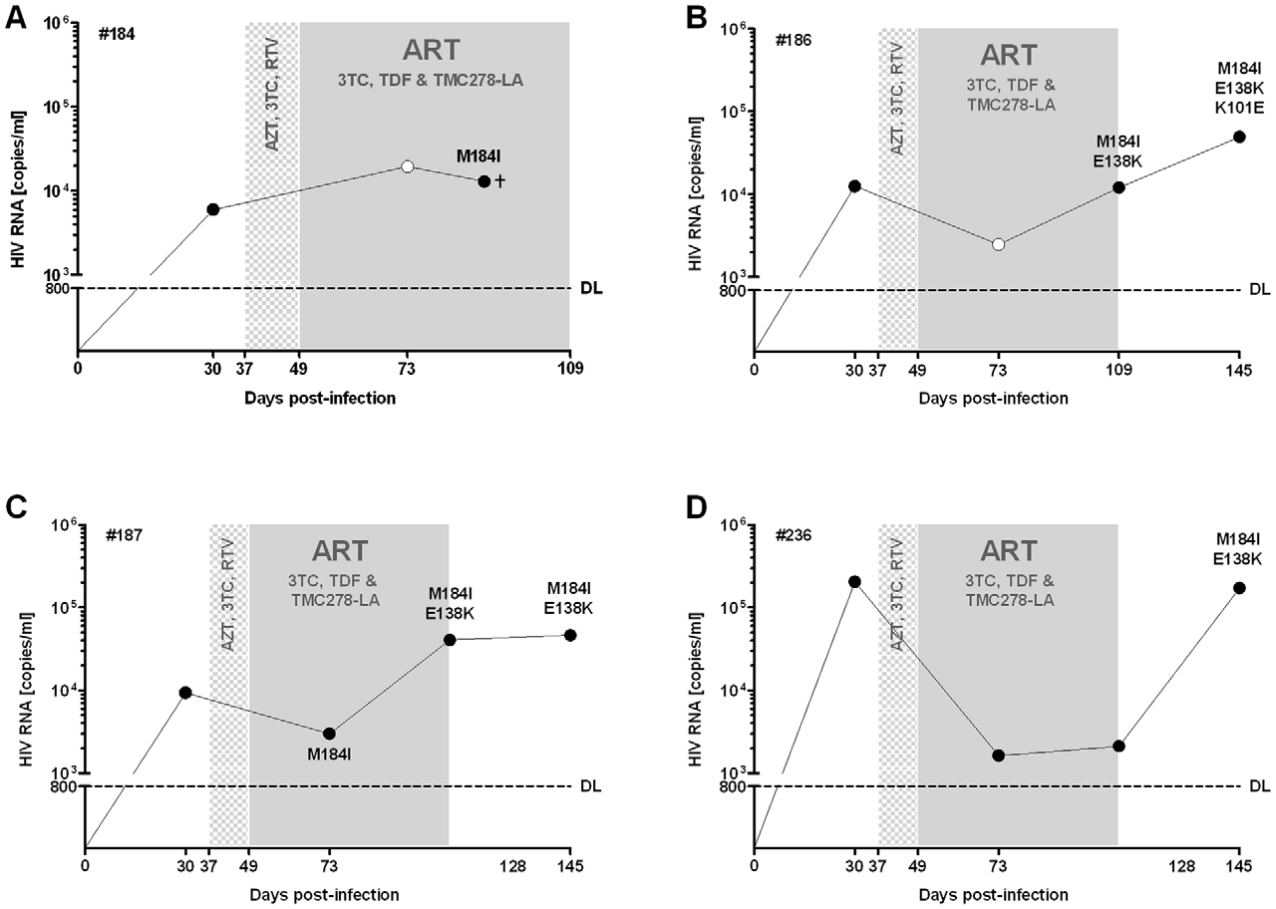
Humanized mice with viral failure under ART with 3TC, TDF and TMC278-LA show consecutive or simultaneous emergence of the key mutation to 3TC or TMC278. A), B), C) and D) show the four mice with emergence of resistance under ART. The black dots indicate that resistance testing had been done, white dots no resistance testing done.

All mice on ART with 3TC, TDF and TMC278-LA which experienced viral failure revealed the consecutive or simultaneous emergence of the prototype 3TC mutation M184I and the TMC278 mutation E138K (Fig. 3 and Table 1). All but one mouse showed viral failure when treated with TMC278-LA monotherapy. Of those mice with viral failure, all but one had the E138K mutation either alone or with the M184I mutation (Table 1). It is unknown if minor drug variants or mutations outside the amplified RT region might explain the lack of any TMC278 resistance mutation detected in the one mouse scored fully susceptible to TMC278 [23].

**Table 1:** Emergence of resistance in the mice under monotherapy with TMC278-LA.

Mouse (Identifier)	day 30 (baseline)	day 73 (under ART with 3TC, TDF, TMC278-LA)	day 128 (38 days under TMC278-LA alone)	day 150 (22 days after interruption of TMC278-LA)
# 192**	S*	n.d.¶	S	S
# 242	S	n.d.	n.d.	S
# 232	S	n.d.	E138K	E138K
# 190***	S	M184V	n.d.	M184I/E138K
# 189	S	S	M184I/K101E/E138K	M184I/K101E/E138K
# 191***	S	M184I	M184I/E138K	M184I/E138K
# 224***	S	M184I	n.d.	M184I/E138K

* S = susceptible (wildtype strain).

** #192 showed suppressed HIV RNA under TMC278-LA monotherapy.

*** #191, 224 showed viral failure under the ART regimen of 3TC, TDF and TMC278-LA. #190 gave a positive signal for HIV RNA but below the limit of detection (<800 copies/ml)

#221, 245 only baseline analyses have been done, and therefore data from these mice were not integrated in the table.

¶#n.d. = not done.

### ART with two long-acting drugs (TMC278-LA and TMC181-LA) effectively treats HIV-infected hu-mice

**Figure 4:**
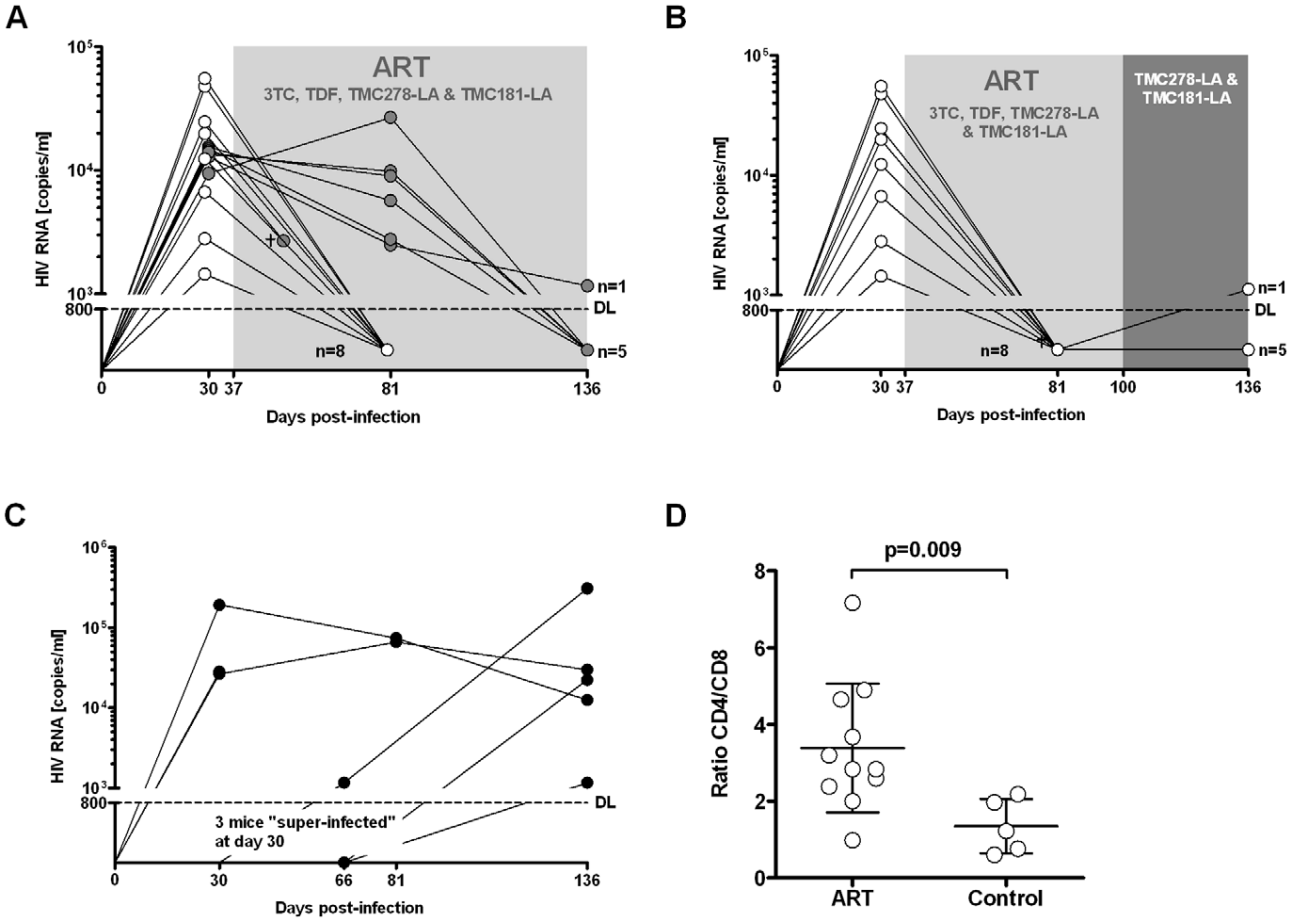
Two long-acting drugs, TMC278-LA (160 mg/kg) and TMC181-LA (400 mg/kg), are highly effective as maintenance therapy. (A) Response to a quadruple ART consisting of 3TC, TDF, TMC278-LA and TMC181-LA over a treatment period of 150 days (the black circles identifies the mice which remained over the entire time on ART (n = 7), the white circles identify the mice which were subsequently switched to a treatment with double long-acting drugs (see (B)). (B) Sustained successful suppression of HIV RNA after switching mice with suppressed HIV RNA under quadruple ART to a treatment with double long-acting drugs (n = 8). (C) Mock-treated HIV infected mice (n = 5). (D) CD4+ T-cells as determined by the CD4/CD8 cell ratio in all treated mice (ART and double long-acting drugs) and mock-treated mice at the end of the experiment.

Next, we sought to determine if a simplified ART of two long-acting drugs interfering at different steps in the HIV replication cycle is effective as maintenance therapy to keep HIV RNA under the detection limit. Initial ART was with TDF, 3TC, TMC278-LA and TMC181-LA. In mice with documented HIV RNA below detection limit after 44 days of ART, we simplified the regimen as planned. The other mice that still showed a marked viremia were continued on the quadruple ART. The dual ART consisting of TMC278-LA and TMC181-LA given once weekly s.c. was really potent: all but one mouse (viral blip) had undetectable HIV RNA (Fig. 4B). Note, that the HIV RNA of all mice which were maintained on the quadruple ART became negative at 99 days after start of treatment (Fig. 4A). This variable decay rate in response to ART mirrors the distinct decay rate of HIV in patients starting ART. As expected the CD4/CD8 T-cell ratio was significantly higher in the mice with suppressed HIV RNA than in control mice (Fig. 4D).

### ART-treated HIV-infected hu mice had HIV RNA under 50 copies/ml but detectable cell-associated HIV DNA

The limited amounts of blood in any running experiment held the sensitivity of the Amplicor Roche® to 400–800 copies/ml. Although it is unlikely, this detection limit does not exclude low viral replication in the ART treated mice. The HIV RNA measurement based on the final bleeding of the mice treated either with the dual ART or the quadruple ART (3TC, TDF, TMC-278LA, TMC181-LA) revealed that 8/10 mice had fewer than 60 copies/ml (Table 2), which emphasizes the efficacy of the ART in the current setting.

**Table 2:** HIV RNA load at the terminal bleeding in mice on ART or on double long-acting drugs.

	Mice on quadruple ART					Mice on double-long acting drugs				
Mouse identification number	#411	#412	#417	#432	#459	#402	#413	#415	#457	#466
HIV RNA	n.d.*	n.d.	152	n.d.	<40	<60	502	n.d.	n.d.	n.d.
Detection limit [copies/ml]	40	40	40	40	40	60	40	60	60	40

* n.d.  =  non-detectable.

- Humanized mice were sacrificed 151 days after HIV infection and 114 days after starting ART or double long-acting drugs.

- Detection limit: the volume of plasma available was slightly different for the mice euthanized and thus the lower detection limit varied accordingly between 40 and 60 copies/ml.

**Figure 5:**
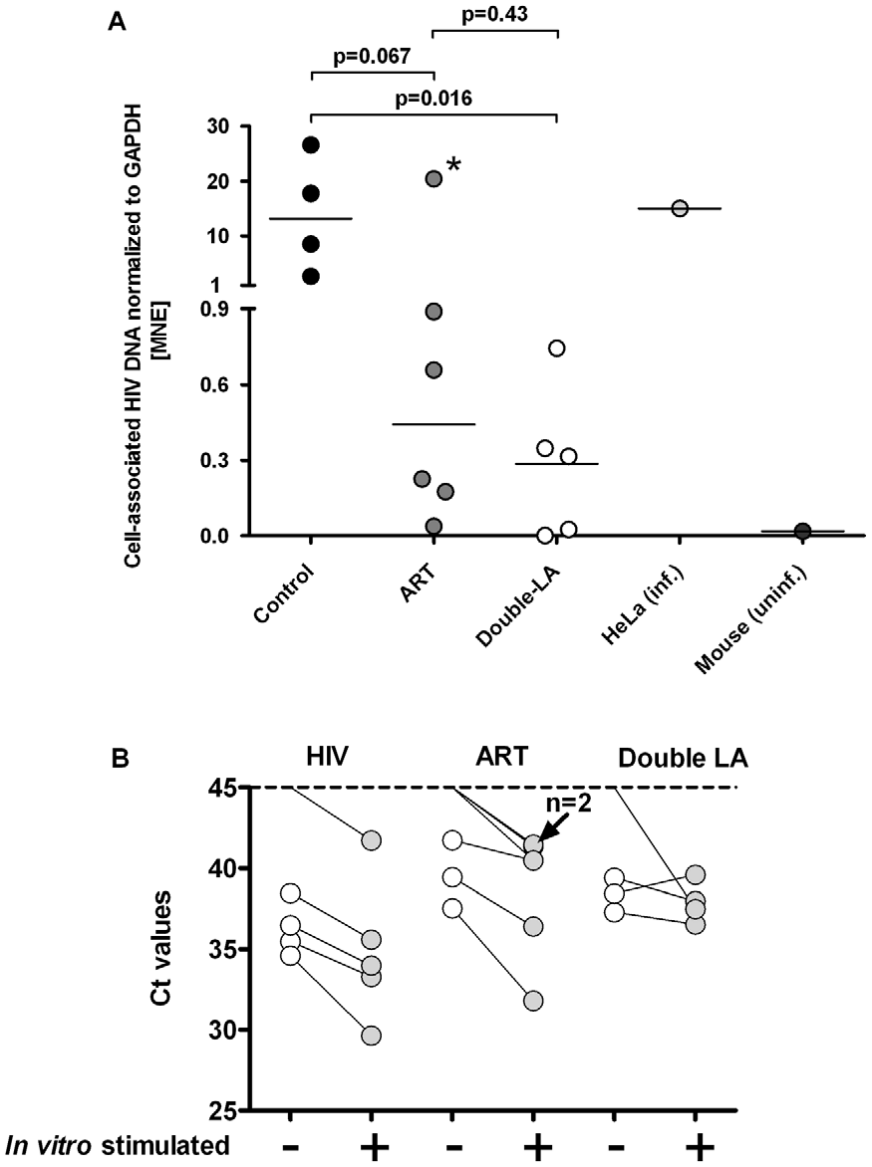
Recovery of cell-associated HIV DNA (A) and increase of HIV mRNA transcripts in vitro from splenic tissue obtained from HIV-infected mice with suppressed HIV RNA following activation. (A) DNA from infected HeLa cells (HeLa inf) and from the spleen of HIV-infected mice served as positive controls, DNA from an uninfected humanized mouse (uninf) served as negative control. The specimens of the treated and HIV-infected mice were from the experiments investigating the antiviral potency of the double long-acting drugs; (MNE = mean normalized expression). B) Splenic tissue specimens from either HIV infected ART naïve hu mice (HIV), ART treated mice (ART) or mice treated with the two long acting drugs (Double-LA) were subjected to mitogens (PMA, PHA) in concert with anti-CD3/28 and IL-7. 18 hours later RNA was extracted and real-time PCR done for quantifying HIV Gag transcripts. Specimens of two mice which were treated with double LA drugs did not show any HIV transcript at all (data not shown in the graph); in five mice we did not detect any HIV transcripts prior to stimulation. The real-time PCRs were done in duplicates. *this specimen is from a mouse (#417) with detectable HIV RNA at the time of euthanization (see Table 2).

In the mice from the second experiment (i.e., mice as shown in Fig. 4), we determined if cell-associated HIV DNA was detectable in splenocytes. Using a real-time PCR specific for YU-2, this was indeed the case in 13/15 mice (Fig. 5A); in all untreated mice, we detected levels of cell-associated HIV DNA higher than in treated ones with the exception of mouse #417. This mouse showed a low-level viremia despite ART that easily explains the relatively high cell-associated HIV DNA. We observed no correlation between cell-associated HIV DNA and engraftment levels or peak viremia.

### In vitro reactivation of HIV transcripts in spleen specimens from hu mice in response to re-activating compounds

In order to examine whether latently infected cells exist in hu mice, we examined unspliced HIV Gag mRNA transcripts in spleen specimens from HIV-infected mock- and vero-treated mice prior and after *in vitro* reactivation. Since we were limited in spleen specimens, we opted for a combination of mitogens in concert with anti-CD3/CD28 and IL-7 to increase our chances of successful reactivation. The short term assay we used as well as the anti-apoptotic effect of IL-7 was certainly beneficial for counteracting any toxic effects due to the application of such a cocktail. Indeed, we did not observe any toxic effects 18 h after adding this cocktail when we harvested the tissue for quantifying HIV mRNA transcripts. As previously reported [16], [17], [18], we observed a clear reactivation of HIV Gag transcripts after stimulation (Fig. 5B). We did not isolate the human CD4+ cells to perform *in vitro* reactivation studies – however, we have no reason to assume that the cell subset harbouring latently HIV will differ from the results published [16], [17], [18].

## Discussion

We sought to determine if hu mice recapitulate key features of HIV infection and treatment and to assess the value of long-acting anti-HIV drugs for treating HIV infection. We found that i) conventional ART with two NRTIs and a NNRTI efficiently suppressed HIV viral load and allowed recovery of the immune system; ii) cell-associated HIV DNA was still present in those mice, and interruption of ART resulted in viral rebound; *in vitro* reactivation of spleen specimens from successfully treated mice yielded increased number of HIV mRNA transcripts as compared to baseline; and iii) simplification of ART with two long-acting drugs kept HIV RNA suppressed. Thus, HIV infection in hu mice mirrors key features of HIV infection in humans, including high titer viremia in untreated mice, suppression of HIV RNA when treated with ART but emergence of resistance when treated with insufficient regimens, viral rebound after treatment interruption, and recovery of CD4+ T cells under ART. Thus, hu mice are a highly valuable animal model to assess the antiviral potency of new compounds or novel strategies to eradicate latent HIV.

Liv/thy SCID hu mice have been used before to investigate novel compounds for their anti-HIV activity [5], [24]. However, detailed PK data were mostly lacking, thus making it difficult to interpret the antiviral potency of the compounds. Furthermore, conventional liv/thy SCID hu mice lack peripheral T cells [3]. Thus, SCID-hu Thy/Liv mice were rarely used to study compounds to treat disseminated HIV infection. Most studies administered the drugs either before or immediately after the HIV challenge [25], and that recapitulates post- or pre-exposure prophylaxis where less potent regimens are already effective.

The new generation of hu mice displays an elaborated lymphoid-like system of human origin [6] and is highly susceptible to HIV infection. However, studies with these mice lacked data about their value for standardized pre-clinical testing. Five studies [14], [15], [16], [17], [18] reported the efficacy of ART in HIV-infected mice. In these studies, ART was started within 3 weeks after HIV infection (i.e., rather early after HIV challenge). In the first study, HIV was suppressed in 3/6 mice by i.p. ART, and HIV recurred when treatment was interrupted [15]. Surprisingly, a second study did not observe viral rebound after treatment interruption [14]. In subsequent studies, complete response rates were reported with an intensified regimen (i.p. administration of emtricitabine (FTC), TDF, an integrase inhibitor and enfuvirtide) [16] or high doses of ART given i.p [17]. All but one [16] of these studies lack detailed PK data on the administered drugs and data related to long-term administration of ART and to the anti-HIV efficacy of ART in chronically infected mice. Compared to the PK data we generated in mice and considering the therapeutic range in humans, the dosages applied in some of the studies reported are most likely 3–5-fold over the therapeutic range.

For an effective mouse model, long-term ART must be non-toxic and well tolerated by the mice. This requires solid PK data in the therapeutic range for humans. In our experiments, we added the distinct anti-HIV compounds to the food pellet based upon calculating food intake, weight and metabolic rate. We generated PK data in the therapeutic range for 3TC and TDF. The long-acting drugs TMC278-LA and TMC181-LA were injected s.c. once weekly. We identified dosages that resulted in concentrations clearly above the C_target_ 1 week after its administration. Notably, plasma levels of NRTIs approximate only the concentration of the anti-HIV active intracellular tri-phosphorylated compounds. Since the half-life of the active moiety is longer than from the parental compound [26], we were confident that the dosages would be efficacious for treating disseminated HIV infection in our model. Indeed, this was the case. We observed HIV RNA suppression in 79% in the first and 100% in the second experiment.

From the PK data, the mice in the first experiment were treated for the first 2 weeks with only effective dosages of 3TC and AZT, a dual therapy insufficient to suppress HIV; RTV plasma levels were substantially below the therapeutic range. Furthermore, AZT which was toxic for the mice resulted in decreased food uptake that was reflected by substantial weight loss and sub-therapeutic dose levels. The emergence of drug-resistant HIV was a logical consequence of insufficient ART plasma levels. The observed M184I mutations in our study are the most prevalent among TDF/FTC-treated HIV individuals. Furthermore, we selected the TMC278 E138K resistance–associated mutation in our mouse model. These findings demonstrate that our model consistently reproduced the Phase III trial results of the ECHO & THRIVE studies, which showed that E138K and M184I combination was the most observed resistance associated mutations in patients treated with TMC278 and co-formulated TDF/FTC [27], [28]. Indeed, our data indicate that the M184I mutation precedes the E138K mutation; this suggests that patients with archived HIV strains with the M184I are especially prone to viral failure with a subsequent triple compound–based regimen with 3TC or FTC and TMC278. This mimics what happens in patients with poor adherence and emphasizes the preclinical proof-of concept value of this mouse model for HIV infection.

HIV-infected mice displayed a distinct response rate to ART (i.e., around 50% of all mice under quadruple therapy had an undetectable HIV RNA at day 44, the other 50% at day 99) (Fig. 4A). Since we were limited in blood draws, we have no detailed data about the viral decay in these sets of mice. Notably, we observed a rapid drop of HIV RNA in a majority of HIV-infected mice within 10 days after initiation of ART when we took blood in short intervals (unpublished data). In any case, the response rate to ART may vary in HIV-infected hu mice similar to the variable response observed in HIV-infected individuals starting ART. It would be therefore sensible to perform HIV RNA measurements over at least 2 months to document ART response or failure.

In our experiments, blood draws yielded small amounts, but terminal bleeds yields larger blood volumes, which are a critical determinant for the sensitivity of the Amplicor Roche®. Indeed, the larger blood volumes documented the success of ART with HIV RNA copy numbers below 60/ml in most mice, thus excluding that low-level viremia was still ongoing in the successfully treated animals.

Interrupting ART resulted in prompt viral rebound in all mice. The mice used for that purpose successfully responded to ART and were treated for a longer period of time to assure the decay of potentially low-replicating cells. We also found cell-associated HIV proviral DNA in untreated and successfully treated mice, re-enforcing the fact that HIV generates a latent reservoir in HIV-infected hu mice. Besides, cell-associated HIV proviral DNA levels in untreated mice were higher than in treated ones, similar to the case in humans [29]. Similarly to findings reported [16], [17], [18], we observed a clear increase of HIV transcripts when splenic tissue from HIV infected mice with suppressed HIV RNA was stimulated. These findings are promising for using hu mice for studying the latent reservoir and in particular for studying approaches to eradicate it. We observed no correlation between cellular HIV-associated proviral DNA and engraftment level or peak viremia. These data show that hu mice represent a model to study latency and potentially novel treatment strategies. Viral rebound or its absence *in vivo* will be a definitive end point of novel eradication strategies aiming to cure HIV infection.

We also report the use of our HIV hu mouse model in the study of TMC278 and TMC181 in a galenic formulation that results in plasma levels above the C_target_ for at least 1 week after administration (long-acting drugs) [30], [31]. TMC278 corresponds to the recently approved NNRTI, rilpivirine [32]. Note that TMC181-LA is a prototype HIV long-acting protease inhibitor; however, the compound is not itself a candidate for clinical development. We tested TMC278-LA as adjunct to the backbone of 3TC and TDF for treating disseminated HIV infection and TMC278-LA alone or in combination with TMC181-LA for maintenance therapy in mice with suppressed HIV RNA. As expected, TMC278-LA was very potent in combination with the NRTI backbone (see above). However, TMC278-LA as monotherapy was not sufficient to maintain HIV suppression. Indeed, all mice showed a viral breakthrough. This prompt viral breakthrough is consistent with the clinical experience in humans: the NNRTI class must be given with a potent backbone [33]. Indeed, due to the long half-life of efavirenz (EFV) and nevirapine (NVP), as opposed to other anti-HIV drugs, interruption of ART containing either EFV or NVP at once results in a monotherapy with the risk of the emergence of NNRTI-resistant strains [34], [35]. This clinical observation documents the relatively low genetic barrier of NNRTIs when given alone. In contrast, TMC278-LA in concert with TMC181-LA was highly efficient with a response rate close to 100% over time. In fact, clinical trials are under way to examine simplification of ART (e.g., combination of the protease inhibitor atazanavir and the integrase inhibitor raltegravir) [36]. Thus, this HIV mouse model would be superbly suited for a pre-clinical proof-of-concept of novel ART strategies (e.g., nucleoside sparing regimens, long-acting drugs).

### Conclusions

In summary, we present data of ART responses/failures in larger number of mice that eventually position this hu mouse model as key tool in the evaluation of novel treatment strategies and latency. Hu mice, indeed, recapitulate central steps in HIV infection, including high-titer viral dissemination, response to ART, viral failure in the case of non-adherence and very importantly viral rebound after ART interruption. Viral rebound after interruption of ART points clearly to a latent reservoir of HIV. This model will be crucial when testing compounds for activating the latent reservoir aiming to eradicate and eventually cure HIV. We underscore this statement by documenting the value of long-acting anti-HIV drugs for suppressing HIV that might be very effective in certain clinical situations, e.g., PREP, PEP or in patients with poor adherence, as a simplified maintenance regimen, or in patients unable to swallow drugs.

## Materials and Methods

### Ethics statement

All experiments as well as procurement of human cord blood were approved by ethical committees of the University of Zurich and the Federal Veterinary Department and. The experiments were conducted according to local guidelines (TschV, Zurich) and the Swiss animal protection law (TschG). Human cord blood was collected with informed written consent of the parents.

### Generation of hu mice

Immunodeficient NOD/shi-scid/γ_c_null (NOG) [19] mice were reconstituted and infected as described [9]. Briefly, newborn NOG mice were irradiated 1–3 days after birth with 1 Gy and subsequently injected intra-hepatically with 2.5±0.5×10^5^ CD34+ cells. CD34+ cells were isolated from human cord blood with immunomagnetic beads (Miltenyi Biotec) with an yield of 0.5–4×10^6^ CD34+ cells from one donation (purity >90%). CD34+ cells and “non-target" fractions were stored frozen in liquid nitrogen until use. At around 15 weeks after transplantation, the engraftment of human immune cells was checked by staining peripheral blood mononuclear cells for the panhuman marker CD45. In all experiments, mice were randomized into mock- or ART treated groups.

### Generation of food pellets containing anti-HIV drugs

Food pellets were made by mixing 2.5 g of 3TC, TDF and AZT each, and 5 g of RTV with 5 kg of ground protein-rich, vitamin-fortified food (Nafag 3432, Provimi Kliba AG, Switzerland) which was subsequently formed to food pellets and sterilized by gamma-irradiation with 25 kGy. All batches of food pellets were analyzed for the correct amount of drugs admixed by HPLC (see below). Food and tap water were given ad libitum. TMC278-LA and TMC181-LA were generated by wet milling the compounds to get nanosizes and their subsequent formulation with non-ionic surfactants [30], [31]. They were injected s.c. at 160 and 400 mg/kg, respectively.

### HPLC-MS/MS method for measuring levels of TDF and 3TC in plasma and food pellets and TMC278 and TMC181 in plasma

Concentrations of drugs in the plasma and food pellets were determined by a qualified research liquid chromatography and mass spectroscopy (LC-MS/MS) method. For the analysis of diet, food pellets were diluted with water (1∶10) and homogenized. Aliquots of each homogenate (50 µL) were solubilized with methanol (three volumes) and extracted with an identical volume of acetonitrile. Plasma samples (50 µL) were prepared identically as the food pellet homogenates. Plasma and food pellets were quantified using a specific LC-MS/MS method.

LC-MS/MS analysis was carried out on an API-4000 MS/MS (Applied Biosystems), which was coupled to an HPLC system (Agilent). The MS/MS was operated in the positive ion mode with the TurboIonSpray-interface (electrospray ionization) and optimized for the quantification of the compound (MRM transition for TDF: 520.2>270; for 3TC 288>176; for TMC278 367.2>224 and for TMC181 585.2>429).

The calibration range was flexible and depended on the study design. The limit of quantification was 0.5–10 ng/ml, depending on the compound. The accuracy (intra-batch accuracy for independent QC samples) was 80–120% of the nominal value over the entire concentration range of the samples.

### HIV infection and ART

Mice were infected i.p. with HIV YU-2, 1×10^6^ tissue-culture infectious dose_50_ (TCID_50_) per mouse. TCID_50_ was determined in human CD8+T cell depleted PBMC from three donors which were stimulated by PHA and anti-CD3 beads (Dynal). HIV RNA plasma levels were measured by RT-PCR (AmpliPrep/COBAS TaqMan HIV-1 Test, Roche) at various times after infection.

Mice were monitored three times a week for symptoms or signs of adverse events, according to a standard score sheet.

### Flow cytometry

Human cells, T cells and B cells were measured by flow cytometry of white blood cells stained for human CD45-APC, CD4-PerCP-Cy5.5, CD8-PB, and CD19-PE-Cy7 (all from BD Biosciences).

### qPCR analysis of mouse organ samples

DNA and RNA from half of a spleen were extracted simultaneously with the AllPrep DNA/RNA Kit (Qiagen). DNA qPCR was as described [37], using HotStarTaq Master Mix (Qiagen), 1 μM of each primer and 0.1 μM FH probe. Experiments were done in duplicate with the real-time thermocycler IQ5 (BioRad) and as cycling profile: 95°C 15 min, 60× (95°C 5 s, 55°C 5 s, 60°C 40 s). The following oligonucleotides were used for HIV gag gene: mf319tq (probe): FAM5′-TGC AGC TTC CTC ATT GAT GGT-3′TAMRA [38], ts5′gag (sense): 5′-CAA GCA GCC ATG CAA ATG TTA AAA GA-3′ [37] and skcc (antisense) (5′-TAC TAG TAG TTC CTG CTA TGT CAC TTC C-3′ [39]. The following oligonucleotides were used for the reference gene GAPDH: mf70tq (probe): FAM5′-AAG GTC GGA GTC AAC GGA TTT GGT CGT-3′TAMRA, mf45 (sense) 5′-TCG ACA GTC AGC CGC ATC TT-3′ and mf46 (antisense) 5′-GGC AAC AAT ATC CAC TTT ACC AG-3′. Mean normalized expression (MNE) was calculated with qbasePLUS (version 2.0, Biogazelle). The following oligonucleotides were used for HIV tat/rev gene mf84-YU-2 (sense) 5′-ACA GTC AGA CTC ATC AAA GTT CTC TAT CAA AGC A-3′ [37], mf226tq (probe): FAM5′-AGG GGA CCC GAC AGG CCC-3′TAMRA [37] and mf83 (antisense): 5′-GGA TCT GTC TCT GTC TCT CTC TCC ACC-3′ [37].

Since mice were infected with HIV-1 YU-2, the plasmid encoding YU-2 was used as standard in the PCR reactions with a detection limit down to 5 copies/reaction.

### Reverse transcription

RNA was DNase treated using DNA-free kit (Ambion). For reverse transcription random hexamer primers (Operon Technologies) and SuperScript III reverse transcriptase (Invitrogen) were used. Reverse transcription was performed as described earlier [40]; briefly cDNA synthesis was performed using 10 µl DNase treated RNA in the presence of Ribolock Rnase inhibitor (Fermentas) in a total volume of 50 µl as follows: 60 min at 50°C, 60 min at 55°, 15 min at 70°C and then 1 min on ice. Subsequently 1 µl of RNAseH (NEB) was added to each tube and incubated at 37°C for 20 min. Aliquots were stored at −20°C or used immediately for real-time PCR analysis.

### Ex vivo reactivation

Splenic cells of half of a spleen were thawed and split in two equal parts and then incubated in RPMI containing fetal calf serum (10%), IL-2 (10U/ml), penicillin (5%)/streptomycin (5%) and L-glutamine (5%) at 37°C for 12 hours. Subsequently cells were washed and then cultivated with or without mitogens (PHA at 3 µg/ml, PMA at 10 ng/ml), anti-CD3/CD28 beads (Dynabeads, Invitrogen) and IL-7 at 20 ng/ml. 18 hours later, DNA and RNA were extracted simultaneously with the AllPrep DNA/RNA Kit (Qiagen) which was then used for quantifying the HIV mRNA Gag transcripts.

### Genotyping

Dideoxynucleotide-based sequence analysis was performed as described [41]. Briefly, Dideoxy sequencing reactions were performed on the purified amplicon (ABI Prism Big Dye Terminator Cycle Sequencing Kit, Version 3.1, Applied Biosystems) with a set of eight sequence-specific primers distributed over the PR-RT sequence for both strands: F1, 5′-GAGAGCTTCA GGTTTGGGG-3′; F2, 5′-AATTGGGCCTGAAAATCC-3′; F3, 5′-CCTCCATTCC TTTGGATGGG-3′; F5, 5′-CACTCTTTGG CAACGACCC-3′; R1, 5′-CTCCCACTCAGGAATCC-3′; R3, 5′-CTTCCCAGAA GTCTTGAGTTC-3′; R5, 5′-GGGTCATAAT ACACTCCATG-3′; R6, 5′-GGAATATTGCTGGTGATCC-3′. Reactions were purified with a DyeEx Purification Protocol (Qiagen) and analysed with the ABI3730xl DNA Analyzer (Applied Biosystems). Sequence data files were grouped per sample identifier (ID) and aligned against the reference HXB2 reference sequence by means of the Sequencher TM Program V 4.1.4 (Gene Codes Corp.). A 25% mixture scoring rule (similar to 20% mixture identification by 454 deep sequencing) was used for the electropherogram analysis.

### Calculations and statistics

Statistical analyses were performed using GraphPad Prism5.0 (GraphPad Software). Data were analysed by non-parametric Mann-Whitney test. In all figures, points represent values of individual mice, and lines depict mean values.

## Supporting Information

Figure S1 **Engraftment levels of hu mice before HIV infection.** The mice were checked for engraftment levels at a median age of 132 days (25–75% percentiles: 103–136) as quantified by staining peripheral blood for the panhuman marker CD45. In addition, the percentage of CD4+, CD8+ and CD19+ cells were determined by flowcytometry.
(TIF)

Figure S2 **PK data of AZT and RTV.** (A and B) Plasma levels of AZT and RTV, respectively, over a day of mice on food pellets containing 0.5 mg/g or 1 mg/g food of AZT or RTV, respectively. The mice used for analysis of PK data have been on food pellets containing drugs for around 2 weeks for PK equilibration. The shaded area indicates the therapeutic range as defined in human. The different colours indicate the experiments done with the same food batch.
(TIF)

Figure S3 **AZT at the dose applied was highly toxic.** Mice were 2 weeks on a regimen with AZT as added at 0.5 mg/kg to the food pellets and were subsequently euthanized. Extensive laboratory chemistry and hematology work-up was done by the Institute of Clinical Chemistry and the Division of Hematology, USZ.
(TIF)

## References

[1] Walensky RP, Paltiel AD, Losina E, Mercincavage LM, Schackman BR (2006). The survival benefits of AIDS treatment in the United States.. J Infect Dis.

[2] Heeney JL, Dalgleish AG, Weiss RA (2006). Origins of HIV and the evolution of resistance to AIDS.. Science.

[3] McCune JM, Namikawa R, Kaneshima H, Shultz LD, Lieberman M (1988). The SCID-hu mouse: murine model for the analysis of human hematolymphoid differentiation and function.. Science.

[4] Namikawa R, Kaneshima H, Lieberman M, Weissman IL, McCune JM (1988). Infection of the SCID-hu mouse by HIV-1.. Science.

[5] Bonyhadi ML, Kaneshima H (1997). The SCID-hu mouse: an in vivo model for HIV-1 infection in humans.. Mol Med Today.

[6] Traggiai E, Chicha L, Mazzucchelli L, Bronz L, Piffaretti JC (2004). Development of a human adaptive immune system in cord blood cell-transplanted mice.. Science.

[7] Jiang Q, Zhang L, Wang R, Jeffrey J, Washburn ML (2008). FoxP3+CD4+ regulatory T cells play an important role in acute HIV-1 infection in humanized Rag2−/−gammaC−/− mice in vivo.. Blood.

[8] Shultz LD, Ishikawa F, Greiner DL (2007). Humanized mice in translational biomedical research.. Nat Rev Immunol.

[9] Baenziger S, Tussiwand R, Schlaepfer E, Mazzucchelli L, Heikenwalder M (2006). Disseminated and sustained HIV infection in CD34+ cord blood cell-transplanted Rag2−/−gamma c−/− mice.. Proc Natl Acad Sci U S A.

[10] Berges BK, Wheat WH, Palmer BE, Connick E, Akkina R (2006). HIV-1 infection and CD4 T cell depletion in the humanized Rag2−/−gamma c−/− (RAG-hu) mouse model.. Retrovirology.

[11] Watanabe S, Terashima K, Ohta S, Horibata S, Yajima M (2007). Hematopoietic stem cell-engrafted NOD/SCID/IL2Rgamma null mice develop human lymphoid systems and induce long-lasting HIV-1 infection with specific humoral immune responses.. Blood.

[12] Zhang L, Kovalev GI, Su L (2007). HIV-1 infection and pathogenesis in a novel humanized mouse model.. Blood.

[13] Gorantla S, Sneller H, Walters L, Sharp JG, Pirruccello SJ (2007). Human immunodeficiency virus type 1 pathobiology studied in humanized BALB/c-Rag2−/−gammac−/− mice.. J Virol.

[14] Sango K, Joseph A, Patel M, Osiecki K, Dutta M (2010). Highly active antiretroviral therapy potently suppresses HIV infection in humanized Rag2−/−gammac−/− mice.. AIDS Res Hum Retroviruses.

[15] Choudhary SK, Rezk NL, Ince WL, Cheema M, Zhang L (2009). Suppression of human immunodeficiency virus type 1 (HIV-1) viremia with reverse transcriptase and integrase inhibitors, CD4+ T-cell recovery, and viral rebound upon interruption of therapy in a new model for HIV treatment in the humanized Rag2−/−{gamma}c−/− mouse.. J Virol.

[16] Choudhary SK, Archin NM, Cheema M, Dahl NP, Garcia JV (2012). Latent HIV-1 infection of resting CD4 T cells in the humanized Rag2/ gammac/ mouse.. J Virol.

[17] Denton PW, Olesen R, Choudhary SK, Archin NM, Wahl A (2012). Generation of HIV latency in humanized BLT mice.. J Virol.

[18] Marsden MD, Kovochich M, Suree N, Shimizu S, Mehta R (2012). HIV latency in the humanized BLT mouse.. J Virol.

[19] Ito M, Hiramatsu H, Kobayashi K, Suzue K, Kawahata M (2002). NOD/SCID/gamma(c)(null) mouse: an excellent recipient mouse model for engraftment of human cells.. Blood.

[20] Watanabe S, Ohta S, Yajima M, Terashima K, Ito M (2007). Humanized NOD/SCID/IL2Rgamma(null) mice transplanted with hematopoietic stem cells under nonmyeloablative conditions show prolonged life spans and allow detailed analysis of human immunodeficiency virus type 1 pathogenesis.. J Virol.

[21] Reagan-Shaw S, Nihal M, Ahmad N (2008). Dose translation from animal to human studies revisited.. FASEB J.

[22] Schrijvers R, Desimmie BA, Debyser Z (2011). Rilpivirine: a step forward in tailored HIV treatment.. Lancet.

[23] Vandamme AM, Camacho RJ, Ceccherini-Silberstein F, de Luca A, Palmisano L (2011). European recommendations for the clinical use of HIV drug resistance testing: 2011 update.. AIDS Rev.

[24] Goldstein H, Pettoello-Mantovani M, Katopodis NF, Kim A, Yurasov S (1996). SCID-hu mice: a model for studying disseminated HIV infection.. Semin Immunol.

[25] Stoddart CA, Bales CA, Bare JC, Chkhenkeli G, Galkina SA (2007). Validation of the SCID-hu Thy/Liv mouse model with four classes of licensed antiretrovirals.. PLoS ONE.

[26] Bazzoli C, Jullien V, Le Tiec C, Rey E, Mentre F (2010). Intracellular Pharmacokinetics of Antiretroviral Drugs in HIV-Infected Patients, and their Correlation with Drug Action.. Clin Pharmacokinet.

[27] Cohen CJ, Andrade-Villanueva J, Clotet B, Fourie J, Johnson MA (2011). Rilpivirine versus efavirenz with two background nucleoside or nucleotide reverse transcriptase inhibitors in treatment-naive adults infected with HIV-1 (THRIVE): a phase 3, randomised, non-inferiority trial.. Lancet.

[28] Molina JM, Cahn P, Grinsztejn B, Lazzarin A, Mills A (2011). Rilpivirine versus efavirenz with tenofovir and emtricitabine in treatment-naive adults infected with HIV-1 (ECHO): a phase 3 randomised double-blind active-controlled trial.. Lancet.

[29] Chun TW, Justement JS, Moir S, Hallahan CW, Maenza J (2007). Decay of the HIV reservoir in patients receiving antiretroviral therapy for extended periods: implications for eradication of virus.. J Infect Dis.

[30] Baert L, van't Klooster G, Dries W, Francois M, Wouters A (2009). Development of a long-acting injectable formulation with nanoparticles of rilpivirine (TMC278) for HIV treatment.. Eur J Pharm Biopharm.

[31] van't Klooster G, Hoeben E, Borghys H, Looszova A, Bouche MP (2010). Pharmacokinetics and disposition of rilpivirine (TMC278) nanosuspension as a long-acting injectable antiretroviral formulation.. Antimicrob Agents Chemother.

[32] Miller CD, Crain J, Tran B, Patel N (2011). Rilpivirine: a new addition to the anti-HIV-1 armamentarium.. Drugs Today (Barc).

[33] Camacho R, Teofilo E (2011). Antiretroviral therapy in treatment-naive patients with HIV infection.. Curr Opin HIV AIDS.

[34] Taylor S, Boffito M, Khoo S, Smit E, Back D (2007). Stopping antiretroviral therapy.. AIDS.

[35] Fox Z, Phillips A, Cohen C, Neuhaus J, Baxter J (2008). Viral resuppression and detection of drug resistance following interruption of a suppressive non-nucleoside reverse transcriptase inhibitor-based regimen.. AIDS.

[36] Cordery DV, Hesse K, Amin J, Cooper DA (2010). Raltegravir and unboosted atazanavir dual therapy in virologically suppressed antiretroviral treatment-experienced HIV patients.. Antivir Ther.

[37] Kaiser P, Joos B, Niederost B, Weber R, Gunthard HF (2007). Productive human immunodeficiency virus type 1 infection in peripheral blood predominantly takes place in CD4/CD8 double-negative T lymphocytes.. J Virol.

[38] Althaus CF, Gianella S, Rieder P, von Wyl V, Kouyos RD (2010). Rational design of HIV-1 fluorescent hydrolysis probes considering phylogenetic variation and probe performance.. J Virol Methods.

[39] Christopherson C, Kidane Y, Conway B, Krowka J, Sheppard H (2000). PCR-Based assay to quantify human immunodeficiency virus type 1 DNA in peripheral blood mononuclear cells.. JClinMicrobiol.

[40] Manrique A, Rusert P, Joos B, Fischer M, Kuster H (2007). In vivo and in vitro escape from neutralizing antibodies 2G12, 2F5, and 4E10.. J Virol.

[41] T. Pattery YV, H. De Wolf, D Nauwelaers, K. Van Baelen, M. Van Houtte, P. Mc Kenna, J Villacian (2012). Development and Performance of Conventional HIV-1 Phenotyping (Antivirogram) and Genotype-Based Calculated Phenotyping Assay (virco TYPE HIV-1) on Protease and Reverse Transcriptase Genes to Evaluate Drug Resistance.. Intervirology.

[42] Fletcher CV, Kawle SP, Kakuda TN, Anderson PL, Weller D (2000). Zidovudine triphosphate and lamivudine triphosphate concentration-response relationships in HIV-infected persons.. AIDS.

[43] Chittick GE, Zong J, Blum MR, Sorbel JJ, Begley JA (2006). Pharmacokinetics of tenofovir disoproxil fumarate and ritonavir-boosted saquinavir mesylate administered alone or in combination at steady state.. Antimicrob Agents Chemother.

