# Supplementary figures and images for: Humanized Mice Recapitulate Key Features of HIV-1 Infection: A Novel Concept Using Long-Acting Anti-Retroviral Drugs for Treating HIV-1
Source: PLoS One. 2012 Jun 13;7(6):e38853. doi: 10.1371/journal.pone.0038853 (PMC3374767; doi:10.1371/journal.pone.0038853)

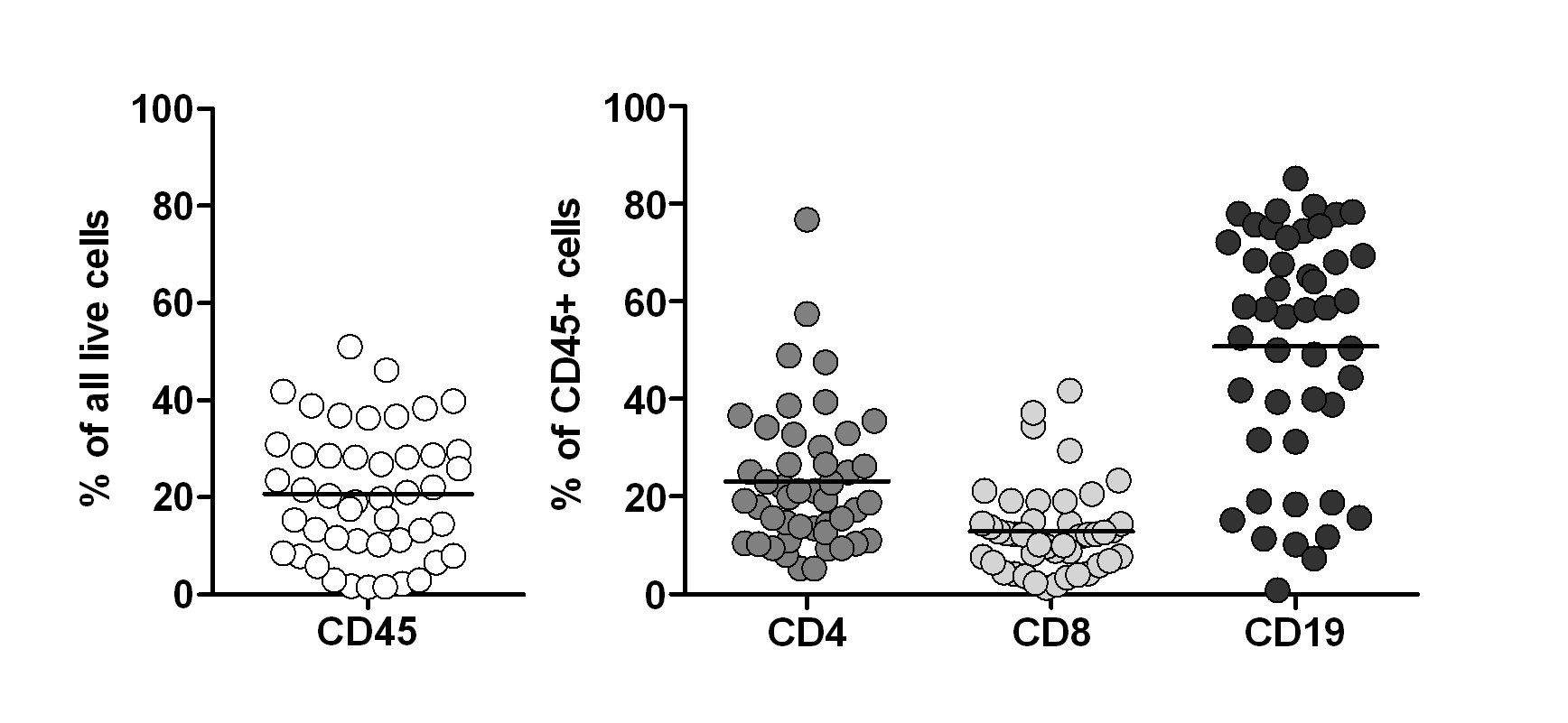

Supplement: Figure S1 — Engraftment levels of hu mice before HIV infection. The mice were checked for engraftment levels at a median age of 132 days (25–75% percentiles: 103–136) as quantified by staining peripheral blood for the panhuman marker CD45. In addition, the percentage of CD4+, CD8+ and CD19+ cells were determined by flowcytometry. (TIF) [file pone.0038853.s001.tif]

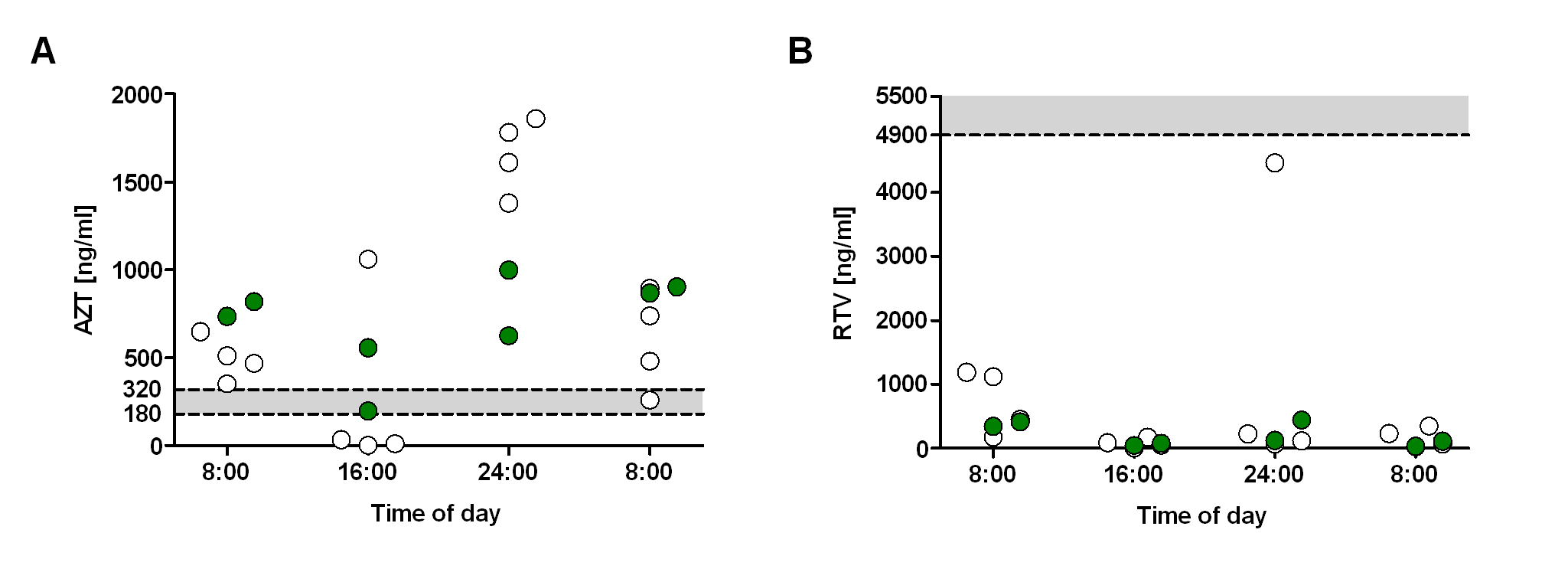

Supplement: Figure S2 — PK data of AZT and RTV. (A and B) Plasma levels of AZT and RTV, respectively, over a day of mice on food pellets containing 0.5 mg/g or 1 mg/g food of AZT or RTV, respectively. The mice used for analysis of PK data have been on food pellets containing drugs for around 2 weeks for PK equilibration. The shaded area indicates the therapeutic range as defined in human. The different colours indicate the experiments done with the same food batch. (TIF) [file pone.0038853.s002.tif]

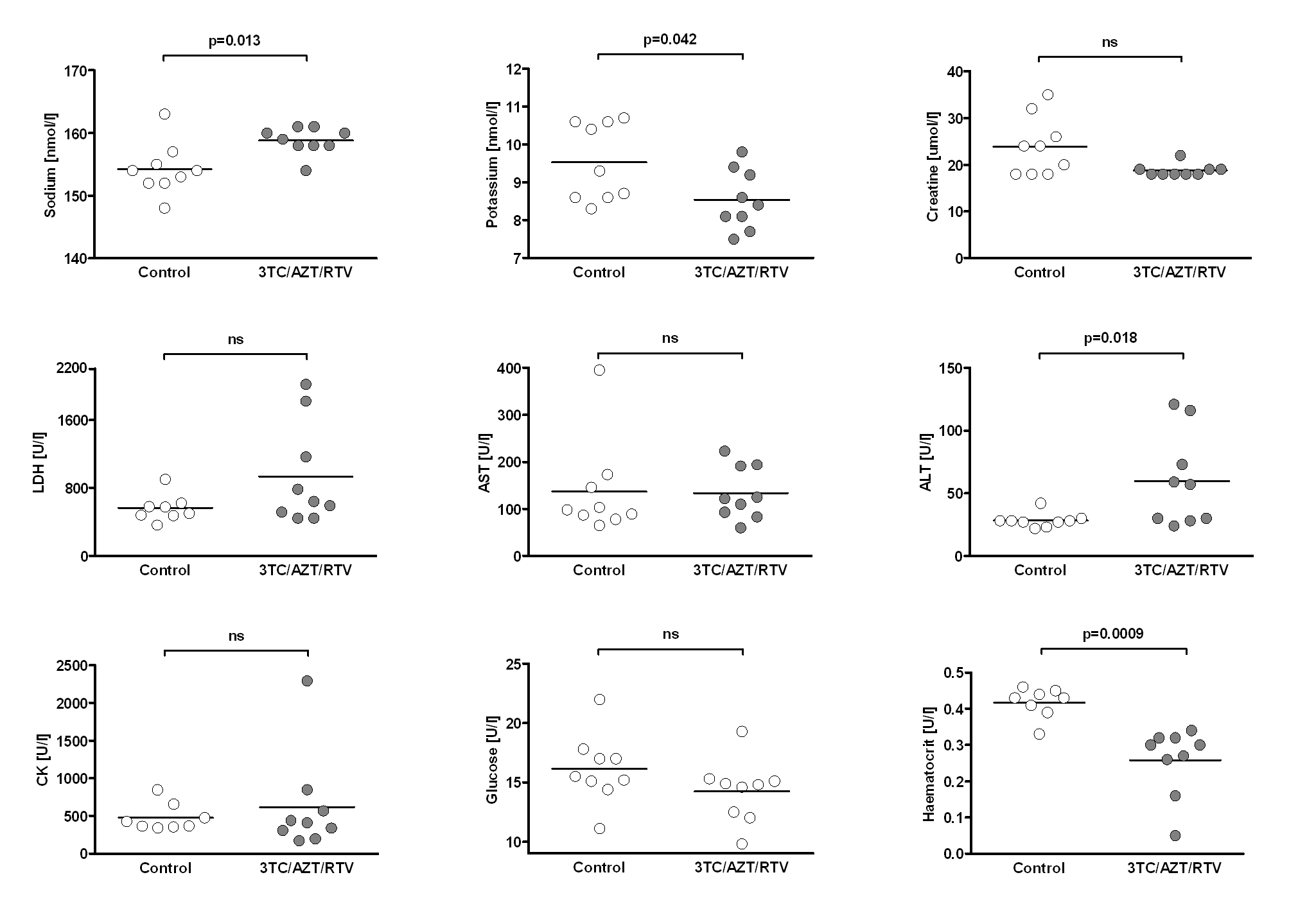

Supplement: Figure S3 — AZT at the dose applied was highly toxic. Mice were 2 weeks on a regimen with AZT as added at 0.5 mg/kg to the food pellets and were subsequently euthanized. Extensive laboratory chemistry and hematology work-up was done by the Institute of Clinical Chemistry and the Division of Hematology, USZ. (TIF) [file pone.0038853.s003.tif]
